# Long-term risk of subsequent ipsilateral lesions after surgery with or without radiotherapy for ductal carcinoma in situ of the breast

**DOI:** 10.1038/s41416-021-01496-6

**Published:** 2021-08-18

**Authors:** Maartje van Seijen, Esther H. Lips, Liping Fu, Daniele Giardiello, Frederieke van Duijnhoven, Linda de Munck, Lotte E. Elshof, Alastair Thompson, Elinor Sawyer, Marc D. Ryser, E. Shelley Hwang, Marjanka K. Schmidt, Paula H. M. Elkhuizen, Grand Challenge PRECISION Consortium, Jelle Wesseling, Michael Schaapveld

**Affiliations:** 1 Division of Molecular Pathology, The Netherlands Cancer Institute-Antoni van Leeuwenhoek Hospital, Amsterdam, The Netherlands; 2 Department of Surgery, The Netherlands Cancer Institute-Antoni van Leeuwenhoek Hospital, Amsterdam, The Netherlands; 3 Department of Research and Development, Netherlands Comprehensive Cancer Organisation, Utrecht, The Netherlands; 4 Department of radiology, Meander Medical Centre, Amersfoort, The Netherlands; 5 Dan L Duncan Comprehensive Cancer Centre, Baylor College of Medicine, Houston, TX USA; 6 Division of Cancer Studies, King’s College London, Comprehensive Cancer Centre, Guy’s Hospital, London, UK; 7 Department of Population Health Sciences, Duke University, Durham, NC USA; 8 Department of Mathematics, Duke University, Durham, NC USA; 9 Department of Surgery, Duke University, Durham, NC USA; 10 Department of clinical genetics, Leiden University Medical Center, Leiden, The Netherlands; 11 Department of radiotherapy, The Netherlands Cancer Institute-Antoni van Leeuwenhoek Hospital, Amsterdam, The Netherlands; 12 Department of pathology, The Netherlands Cancer Institute-Antoni van Leeuwenhoek Hospital, Amsterdam, The Netherlands; 13 Department of Pathology, Leiden University Medical Center, Leiden, The Netherlands; 14 Division of Psychosocial Research and Epidemiology, The Netherlands Cancer Institute-Antoni van Leeuwenhoek Hospital, Amsterdam, The Netherlands

**Keywords:** Radiotherapy, Outcomes research, Breast cancer, Cancer epidemiology

## Abstract

**Background:**

Radiotherapy (RT) following breast-conserving surgery (BCS) for ductal carcinoma in situ (DCIS) reduces ipsilateral breast event rates in clinical trials. This study assessed the impact of DCIS treatment on a 20-year risk of ipsilateral DCIS (iDCIS) and ipsilateral invasive breast cancer (iIBC) in a population-based cohort.

**Methods:**

The cohort comprised all women diagnosed with DCIS in the Netherlands during 1989–2004 with follow-up until 2017. Cumulative incidence of iDCIS and iIBC following BCS and BCS + RT were assessed. Associations of DCIS treatment with iDCIS and iIBC risk were estimated in multivariable Cox models.

**Results:**

The 20-year cumulative incidence of any ipsilateral breast event was 30.6% (95% confidence interval (CI): 28.9–32.6) after BCS compared to 18.2% (95% CI 16.3–20.3) following BCS  +  RT. Women treated with BCS compared to BCS + RT had higher risk of developing iDCIS and iIBC within 5 years after DCIS diagnosis (for iDCIS: hazard ratio (HR)_age < 50_ 3.2 (95% CI 1.6–6.6); HR_age ≥ 50_ 3.6 (95% CI 2.6–4.8) and for iIBC: HR_age<50_ 2.1 (95% CI 1.4–3.2); HR_age ≥ 50_ 4.3 (95% CI 3.0–6.0)). After 10 years, the risk of iDCIS and iIBC no longer differed for BCS versus BCS + RT (for iDCIS: HR_age < 50_ 0.7 (95% CI 0.3–1.5); HR_age ≥ 50_ 0.7 (95% CI 0.4–1.3) and for iIBC: HR_age < 50_ 0.6 (95% CI 0.4–0.9); HR_age ≥ 50_ 1.2 (95% CI 0.9–1.6)).

**Conclusion:**

RT is associated with lower iDCIS and iIBC risk up to 10 years after BCS, but this effect wanes thereafter.

## Introduction

Since the introduction of population-based mammography breast cancer screening in the 1990s, ductal carcinoma in situ (DCIS) comprises ~15% of all newly diagnosed neoplastic breast lesions [1, 2]. DCIS is considered a non-obligate precursor of invasive breast cancer (IBC) and consists of neoplastic epithelial cells confined to the ductal system of the mammary gland. Because of its potential to become invasive, patients diagnosed with DCIS are usually treated for IBC with a mastectomy or with breast-conserving surgery (BCS) often followed by radiotherapy (RT) to the whole breast (RT). DCIS itself, however, is not life-threatening, and these treatment strategies by definition lead to overtreatment for lesions, which would not progress to IBC within the lifespan of that patient [3, 4].

RT as an adjunct to BCS as a treatment for DCIS was evaluated in several clinical trials (NSABP B17, EORTC 10853, SweDCIS, UK/ANZ), and a meta-analysis demonstrated a 15% absolute 10-year risk reduction of both subsequent ipsilateral DCIS (iDCIS) and ipsilateral IBC (iIBC) lesions for BCS + RT versus BCS only, without effect on breast cancer-specific survival and overall survival [5–9]. However, how these trial data translate into a reduction of ipsilateral breast events in large, population-based patient cohorts in the longer term is unclear. We previously showed an absolute risk for iIBC of 15.4% for patients treated with BCS only compared to 8.8% for patients treated with BCS + RT at 15 years after diagnosis in a cohort with nationwide coverage [10]. Importantly, we observed that iIBC risk no longer appeared to differ in the interval beyond 10 years of follow-up when comparing women treated with BCS only to those treated with BCS + RT. Information regarding subsequent in situ lesions was lacking in our previous study. We now assess the very long-term risk of both iDCIS and iIBC after a diagnosis of primary DCIS, extending median follow-up of >5 years, and assess associations with initial DCIS treatment by age at DCIS diagnosis and elapsed time since diagnosis.

## Methods

### Data collection

Our cohort comprises all women diagnosed with primary pure DCIS in the Netherlands between January 1, 1989 and December 31, 2004 [10]. Diagnoses of subsequent iIBC lesions were derived from the Netherlands Cancer Registry (NCR) as well as through linkage of the NCR database with the nationwide registry of histology and cytopathology in the Netherlands (PALGA). Subsequent iDCIS lesions are not registered within the NCR and therefore identification is solely based on pathology reports provided by the PALGA registry. iDCIS was defined as any ipsilateral ductal carcinoma in situ lesion including microinvasive growth <1 mm at least 3 months after diagnosis of the index DCIS; iIBC was defined as any ipsilateral invasive breast lesion (corresponding to DCIS >1 mm microinvasive growth) diagnosed at least 3 months after diagnosis of the index DCIS. Follow-up for both NCR and PALGA has been completed until January 1, 2017. Initial treatment was categorised into three groups: BCS alone (BCS only), BCS with additional whole breast RT (BCS + RT) or mastectomy (independent of subsequent RT). Chemotherapy and endocrine therapy were almost never administered to women with DCIS in the Netherlands during the time of the cohort accrual, and patients who received chemotherapy or endocrine therapy for DCIS were excluded (*n* = 123). For patients treated with mastectomy, information of iDCIS recurrences was not collected, because a priori we expected these to be negligibly low. Intercurrent mastectomies were defined as mastectomies of the ipsilateral breast ≥3 months after primary DCIS diagnosis and applied for other reasons than our events of interest (iDCIS or iIBC) as identified from pathology reports provided by the PALGA registry. As the Netherlands has a universal health care system for all inhabitants, all women diagnosed with DCIS had equal access to treatment. In this paper, subsequent ipsilateral lesions are referred to as ‘recurrence’, although we do not know whether these lesions are biologically related to the primary DCIS or represent independent secondary primaries.

### Statistical analyses

Time at risk started at the date of primary DCIS diagnosis and ended at the date of the first event of interest (iDCIS or iIBC), date of death, emigration or January 1, 2017, whichever came first. If the laterality of a subsequent iDCIS was unknown, this resulted in censoring at the date of iDCIS (*n* = 10). The cumulative incidence of iDCIS, iIBC and the combination of iDCIS and iIBC was estimated using the Aalen–Johanson estimator with death as the only competing risk and emigration as a censoring event. iDCIS or iIBC was not used as a competing event nor as a censoring event when evaluating the risk of the other. In the cumulative incidence analysis for iIBC, an intercurrent mastectomy was included as time-dependent co-variable, in which patients with an intercurrent mastectomy contributed personal time to the mastectomy group from the date of that intercurrent mastectomy. An intercurrent mastectomy (independent of the reason for the mastectomy) is a censoring event in all the iDCIS analyses and in the Cox analyses for iIBC. For the cumulative incidence analyses, Grays’ sample test [11] was used to assess the differences between treatments; intercurrent mastectomies were not taken into account.

Multivariable Cox proportional hazard analysis was used to examine the effects of treatment strategies on iDCIS and iIBC risk. Attained age was used as a timescale. The proportional hazard assumption was assessed using residual-based and graphical methods. Because the hazard ratios (HRs) for treatment were non-proportional with time since treatment, the models for iDCIS and iIBC risk were stratified by time since treatment, using intervals of 0–4, 5–9 and ≥10 years after diagnosis and an interaction term for treatment and time since treatment, using the above intervals, was added to the models [12]. In addition, the HRs for treatment differed with age at diagnosis (*p*_interaction_ < 0.001). Using the Aikake information criterion, the iIBC model demonstrated the best fit when age at DCIS diagnosis was fitted as a dichotomous categorical variable (<50 versus ≥50 years old) and an age–treatment interaction term was added to the model. For iDCIS, the best fit model was achieved by adjusting for age at DCIS diagnosis as a continuous variable. To keep the models for iDCIS and iIBC comparable, we, however, included age as a dichotomous categorical variable (<50 versus ≥50 years old), while also including an age–treatment interaction term, although for iDCIS this age–treatment interaction was non-significant (*p*_interaction_ = 0.06).

The association of histological grade of the primary DCIS with iDCIS and iIBC risks was evaluated only among patients diagnosed in the period 1999–2004, as information on DCIS grade was incomplete before 1999. In the analysis of iDCIS risk among patients diagnosed in 1999–2004, the proportional hazards assumption was not violated and no interaction term for treatment and time since treatment was included and age neither modified the effect of treatment.

All analyses were performed in open source software R version 3.5.1 using the ‘survival’ and ‘etm’ packages [13].

## Results

**Fig. 1:**
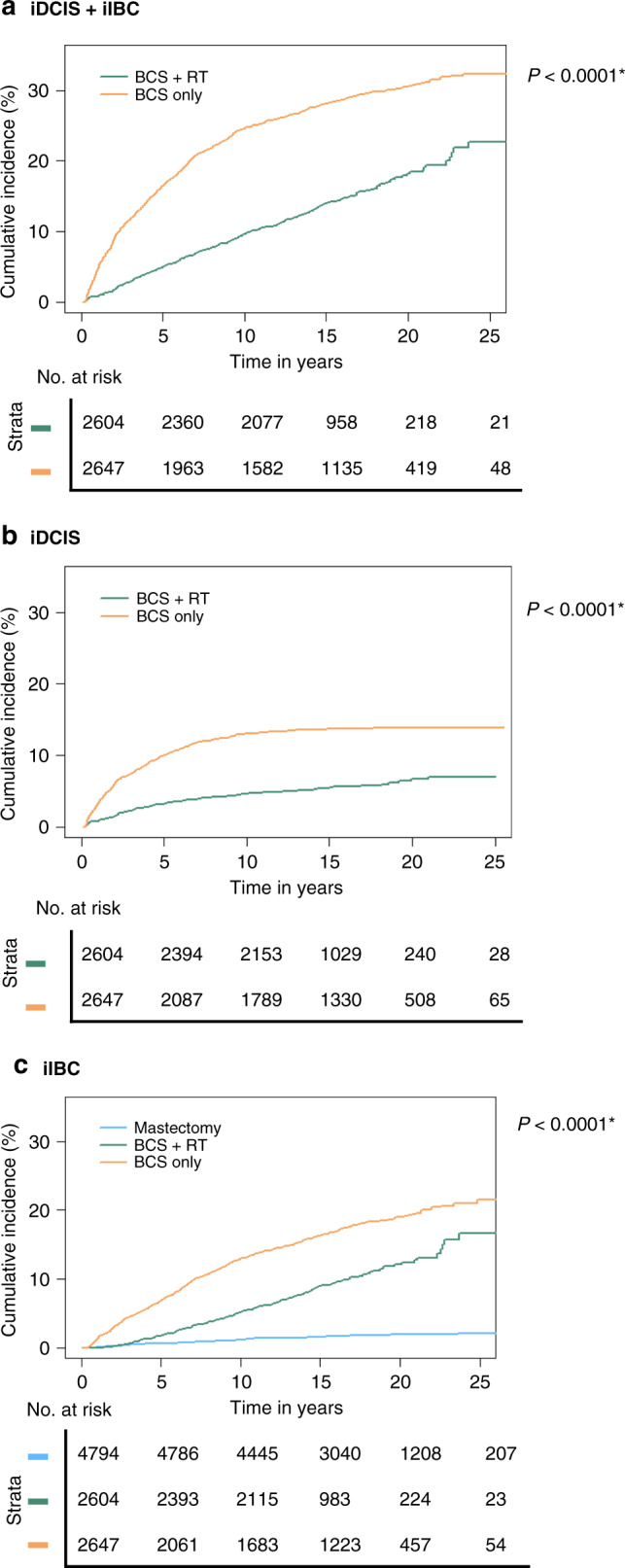
Cumulative incidence with death as the competing risk by treatment strategy. **a** In situ and invasive recurrences, **b** iDCIS only and **c** invasive recurrences only. *P values are based on Grays’ *K* sample test.

The study cohort comprised 10,045 women, of whom 2647 (26%) received BCS only, 2604 (26%) received BCS + RT and 4794 (48%) underwent mastectomy as the primary treatment. Additional patient characteristics are summarised in Table 1. The median follow-up was 15.7 years (interquartile range 9.2–22.3 years). During follow-up, a total of 774 (7.7%) iIBC and 497 (4.9%) iDCIS lesions were identified. The 10- and 20-year cumulative incidence of subsequent ipsilateral breast disease (iDCIS or iIBC) for women treated with BCS only was 24.6% (95% confidence interval (CI): 23.0–26.3) and 30.6% (95% CI 28.9–32.6), respectively, whereas for women treated with BCS + RT, the cumulative incidence was 9.6% (95% CI 8.6–10.8) and 18.2% (95% CI 16.3–20.3) at 10 and 20 years, respectively (Fig. 1). The competing risk, death, varied for the different treatment strategies between 8.7 and 14.7% after 10 years and between 26.8 and 35.2% after 20 years since DCIS diagnosis (Supplementary Fig 1).

**Table 1:** Patient characteristics.

	Initial DCIS treatment			
	BCS only, *N* = 2647	BCS + RT, *N* = 2604	Mastectomy, *N* = 4794	Total, *N* = 10,045
Follow-up (years), median (IQR)	17.0 (9.7–24.4)	14.5 (9.9–19.1)	16.0 (9.0–22.9)	15.7 (9.2–22.3)
Age at DCIS diagnosis (years), median (IQR)	58.9 (43.0–74.8)	57.2 (43.2–71.2)	57.2 (40.6–73.8)	57.6 (41.9–73.3)
Age <50	474 (17.9)	457 (17.5)	1212 (25.3)	2143 (21.3)
Age ≥50	2173 (82.1)	2147 (82.5)	3582 (74.7)	7902 (78.7)
DCIS grade (1999–2004^a^)				
Low (1)	302 (40.9%)	215 (13.7%)	190 (10.2%)	707 (16.9%)
Intermediate (2)	234 (31.7%)	578 (36.7%)	553 (29.7%)	1365 (32.7%)
High (3)	202 (27.4%)	780 (49.6%)	1121 (60.1%)	2103 (50.4%)
Unknown	240	285	342	867
Subsequent iIBC	445	240	89	774
Subsequent iDCIS	352	145	NA	497

*iIBC* ipsilateral invasive breast cancer, *iDCIS* ipsilateral ductal carcinoma in situ, *BCS* breast-conserving surgery, *RT* radiotherapy, *N* number, *IQR* interquartile range, *DCIS* ductal carcinoma in situ, *NA* not available.

^a^Data on grade are presented for patients diagnosed with primary DCIS from 1999 to 2004 (*n* = 5042).

### Subsequent iDCIS risk

Among patients treated with BCS only, 352 iDCIS occurred, compared to 145 iDCIS, among patients treated with BCS + RT. Most iDCIS occurred within the first 10 years of follow-up, with only 19 patients developing a late iDCIS (10 years or more after their initial DCIS diagnosis) after BCS only and 27 after BCS + RT (Supplementary Table 1). For women treated with BCS only, the 10- and 20-year cumulative incidence of iDCIS was 13.0% (95% CI 11.8–14.4) and 13.9% (95% CI 11.6–15.3), respectively, versus 4.6% (95% CI 3.9–5.5) and 6.7% (95% CI 5.5–8.1), respectively, for women treated with BCS + RT (Fig. 1 and Supplementary Table 1).

Women <50 years treated with BCS only had 3.2 times higher HR (95% CI 1.6–6.6) for iDCIS in the first 5 years after diagnosis compared to women treated with BCS + RT, while women ≥50 years treated with BCS only had a 3.6 times higher HR for iDCIS (95% CI 2.6–4.8) than women treated with BCS + RT (Table 2). The HR to develop iDCIS among patients treated with BCS only compared to BCS + RT in the interval 5–9 years after primary DCIS was 2.5 (95% CI 1.1–5.3) for women <50 years and 2.7 (95% CI 1.8–4.1) for women ≥50 years, and risks no longer differed between patients treated with BCS only compared to BCS + RT from 10 years after initial DCIS (Table 2). Women diagnosed between 1999 and 2004 had a slightly lower risk of developing iDCIS compared to women diagnosed between 1989 and 1998 (HR 0.9; 95% CI 0.7–1.0).

**Table 2:** Multivariate Cox analysis to estimate the association of treatment with the risk of subsequent iDCIS and iIBC.

			iDCIS		iIBC	
Age at DCIS (years)	Time since DCIS (years)	Treatment	Events (*N*)/at risk (*N*)	HR (95% CI)	Events (*N*)/at risk (*N*)	HR (95% CI)
<50 (*N* = 2143)	0–5	BCS + RT	15/457	Ref.	17/457	Ref.
		BCS only	57/474	3.2 (1.6–6.6)	33/474	2.1 (1.4–3.2)
		Mastectomy^a^	–	–	19 / 1212	0.4 (0.2–0.6)
	5–10	BCS + RT	8/419	Ref.	22/412	Ref.
		BCS only	12/386	2.5 (1.1–5.3)	23/379	1.0 (0.7–1.5)
		Mastectomy^a^	–	–	13/1161	0.1 (0.1–0.3)
	≥10	BCS + RT	17/383	Ref.	38/363	Ref.
		BCS only	4/353	0.7 (0.3–1.5)	36/331	0.6 (0.4–0.9)
		Mastectomy^a^	–	–	17/1108	0.1 (0.1v0.2)
≥50 (*N* = 7902)	0–5	BCS + RT	69/2147	Ref.	29/2147	Ref.
		BCS only	201/2173	3.6 (2.6–4.8)	137/2173	4.3 (3.0–6.0)
		Mastectomy^a^	–	–	14/3582	0.3 (0.2–0.4)
	5–10	BCS + RT	26/1975	Ref.	63/1981	Ref.
		BCS only	63/1701	2.7 (1.8-4.1)	119/1682	2.1 (1.6–2.8)
		Mastectomy^a^	–	–	9/3314	0.1 (0.1–0.2)
	≥10	BCS + RT	10/1769	Ref.	70/1751	Ref.
		BCS only	15/1436	0.7 (0.4–1.3)	94/1352	1.2 (0.9–1.6)
		Mastectomy^a^	–	–	17/2957	0.1 (0.1–0.1)

*HR* hazard ratio, *95% CI* 95% confidence interval, *Ref*. reference category, *BCS* breast-conserving surgery, *RT* radiotherapy, *iDCIS* ipsilateral ductal carcinoma in situ, *iIBC* ipsilateral invasive breast cancer, *DCIS* ductal carcinoma in situ.

^a^Information regarding mastectomy treatment was not available for iDCIS. Attained age was used as a primary timescale, adjusted for a period of initial DCIS diagnosis (1989–1998 versus 1999–2004) and age at DCIS diagnosis (<50 versus ≥50), including an age–treatment interaction term.

Among all women diagnosed with primary DCIS between 1999 and 2004, women with grade 1 DCIS had half the risk (HR 0.5; 95% CI 0.3–0.8) of iDCIS compared to women with grade 2 lesions (Supplementary Table 2). iDCIS risk did not differ for women with grade 3 lesions compared to those with grade 2 lesions. As 58 patients developed a subsequent iDCIS between 3 and 6 months after DCIS diagnosis, as a sensitivity analysis, we also assessed the risk of iDCIS starting follow-up at 6 months after DCIS diagnosis. Although the HRs slightly increased, particularly for women <50 years, the direction of effects remained the same (see Supplementary Table 3).

### Subsequent iIBC risk

**Fig. 2:**
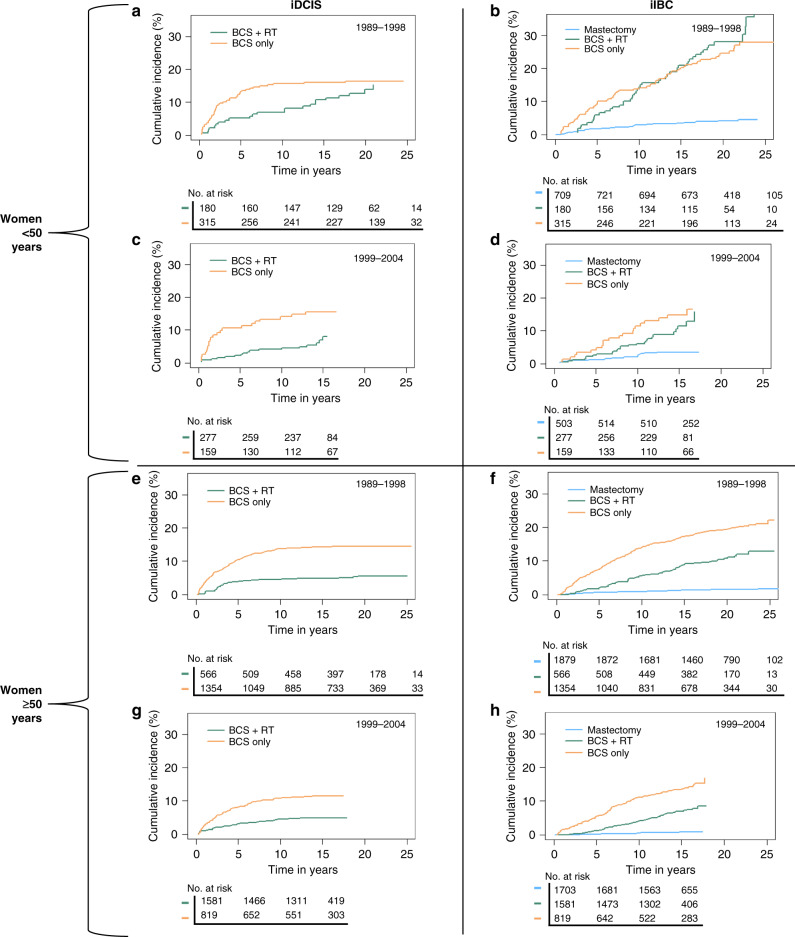
Cumulative incidence of iDCIS and iIBC with death as the competing risk splitted for period and age. Cumulative incidence with death as the competing risk in **a** iDCIS risk of women <50 years diagnosed between 1989 and 1998 for primary DCIS, **b** iIBC risk of women <50 years diagnosed between 1989 and 1998 for primary DCIS, **c** iDCIS risk of women <50 years diagnosed between 1999 and 2004 for primary DCIS and **d** iIBC risk women <50 years diagnosed between 1999 and 2004 for primary DCIS, **e** iDCIS risk of women ≥50 years diagnosed between 1989 and 1998, **f** iIBC risk of women ≥50 years diagnosed between 1989 and 1998, **g** iDCIS risk of women ≥50 years diagnosed between 1999 and 2004 and **h** iIBC risk of women ≥50 years diagnosed between 1999 and 2004.

Among patients treated with BCS only, the 10- and 20-year cumulative incidence of iIBC was 13.9% (95% CI 11.7–14.3) and 19.1% (95% CI 17.5–20.8), respectively. The 10- and 20-year cumulative incidence was 5.2% (95% CI 4.4–6.2) and 12.1% (95% CI 10.5–14.0), respectively, in patients treated with BCS + RT and 1.1% (95% CI 0.9–1.5) and 1.9% (95% CI 1.6–2.4), respectively, in patients treated with mastectomy (Fig. 1 and Supplementary Table 1). Women <50 years diagnosed with DCIS between 1999 and 2004 and treated with BCS + RT showed continuously lower absolute iIBC risks compared to those treated with BCS only (Fig. 2). In contrast, women <50 years diagnosed between the period 1989 and 1998 had approximately similar cumulative incidences after either BCS only or BCS + RT treatment from 10 years or more after DCIS diagnosis.

In women <50 years at DCIS diagnosis, the HR for iIBC was 2.1 times (95% CI 1.4–3.2) higher in the first 5 years after diagnosis among those treated with BCS only compared to women treated with BCS + RT; the HR for iIBC was even 4.3 times (95% CI 3.0–6.0) higher for women ≥50 years treated with BCS only within the first 5 years after treatment compared to BCS + RT (Table 2). The risk of developing an iIBC no longer differ from 5 years after DCIS diagnosis for women <50 years compared to those treated with BCS only or with BCS + RT (HR 1.0; 95% CI 0.7–1.5). While for women ≥50 years, this risk did no longer differ from 10 years after DCIS diagnosis (HR 1.2; 95% CI 0.9–1.6). Women treated with a mastectomy had a much lower risk of developing iIBC compared to women treated with BCS, irrespective of age at diagnosis or time since DCIS treatment (Table 2). Women diagnosed with primary DCIS between 1999 and 2004 had a slightly lower risk of developing iIBC compared to women diagnosed between 1989 and 1999 (HR 0.8; 95% CI 0.6–0.9).

Inclusion of histological grade in the analysis did not affect the association of DCIS treatment with iIBC risk (HR_age ≥ 50_ for BCS only versus BCS + RT in year 1–5: 4.8; 95% CI 2.7–8.5) for a model including grade and 4.8 (95% CI 2.7–8.6) for a model without grade, see Supplementary Table 4 for all estimates) and grade did not modify the association of initial treatment with iIBC risk (*p*_interaction_ = 0.3). As information regarding comorbidities was unavailable, a sensitivity analysis was performed excluding patients ≥70 years, assuming that patients ≥70 years would be most at risk for comorbidities. We found slightly lower risk in the first 5 years after DCIS diagnosis for women ≥50 years treated with BCS only versus BCS + RT (HR 3.6; 95% CI 2.7–4.8) compared to the analysis including all women (HR 4.3; 95% CI 3.0–6.0) (Supplementary Table 5).

## Discussion

In this population-based study among 10,045 women treated for DCIS, we showed that patients treated with BCS only had an absolute risk of 14% to develop iDCIS and of 19% to develop iIBC at 20 years after treatment, while for BCS + RT, these risks were 7% and 12%, respectively. Furthermore, RT is most strongly associated with lower recurrence risks in the first decade after DCIS diagnosis. iDCIS predominantly occurred in the first 10 years after primary DCIS. Finally, the rate of iIBC recurrences did no longer differ between women treated with BCS only versus BCS + RT from 5 years after DCIS diagnosis in women <50 years and from 10 years after DCIS diagnosis in women ≥50 years at primary DCIS.

Our study has some limitations. Firstly, margin status, tumour size and information on initiation and completion of RT were not available for our patients while grade was not routinely scored in the period covered by our study, and, also because of (high) interobserver variability [14, 15], may not have been very reliably assessed. ER and HER2 status are not routinely determined for DCIS lesions in the Netherlands, because it has no therapeutic consequences.

We had no information on comorbidities. In addition, our cohort might not be completely representative of DCIS patients nowadays. The patients in our cohort were diagnosed and treated sometimes decades ago, and pre-operative work-up and RT techniques have evolved over time.

However, the strengths of this study are that it is truly population-based, covering the whole of the Netherlands, with complete information on initial treatment and reliable information on intercurrent mastectomies combined with long and complete follow-up for both in situ IBC and IBC, as well as vital status. Although we defined iDCIS and iIBC as subsequent ipsilateral recurrences ≥3 months after DCIS diagnosis, starting follow-up at 6 months after diagnosis could slightly reduce the chance of counting re-excisions as iDCIS events.

Nonetheless, our data clearly show that late in situ recurrences, ≥10 years after DCIS diagnosis, rarely developed, while the incidence of iIBC continued to rise over time irrespective of initial treatment. This is concordant with the SweDCIS trial [16] and with the Vermont cohort [17], which both reported few iDCIS occurrences after 5 years of follow-up.

An explanation for this plateau in risk of subsequent iDCIS lesions after 10 years might be that recurrent DCIS lesions were less detected after 10 years either due to the fact that patients were discharged from routine surveillance or were no longer within the age range invited for the population breast cancer screening programme. Alternatively, the lack of in situ recurrences after 10 years may reflect the biology of these DCIS lesions, which would suggest that almost all subsequent iDCIS lesions originate from residual primary DCIS. This is supported by the high frequency of clonal relatedness of iDCIS to primary DCIS, reported to be 82% by Waldman et al. [18], while Shah et al. [19] even reported complete clonal relatedness of iDCIS to primary DCIS. Within our consortium, PREvent ductal Carcinoma In Situ Invasive Overtreatment Now (PRECISION) initiative [20], we are conducting genomic studies to determine the clonal relatedness of in situ recurrences to the primary DCIS in order to better understand the relationship between the initial DCIS diagnosis and subsequent breast events.

RT is associated with a lower risk of iDCIS and iIBC, particularly in the first 10 years after the initial DCIS diagnosis. This is in line with a prior meta-analysis that showed that RT reduced the absolute 10-year risk by 15% (28.1% any recurrence in BCS-only group versus 12.9% in BCS + RT group [6]) and with several cohort studies, which all showed that RT reduced breast events after RT in addition to BCS [17, 21–23]. However, our analysis also showed that 10 years or more after DCIS diagnosis, the incidence of new iIBC is approximately similar in the BCS-only and BCS + RT group (Fig. 1 and Supplementary Table 1). This is consistent with the results of Rakovitch et al. [24], who showed lower risks of second breast events with increasing follow-up time after DCIS diagnosis. Since extensive clonal diversity is generated by mutations gradually evolving over time [25], it becomes more likely that newly developed tumours represent an independent second primary tumour >10 years after initial DCIS. However, to our knowledge, the association of follow-up time with clonal relatedness between primary DCIS and subsequent lesions has not yet been assessed. In addition, we cannot exclude the possibility that RT may induce (secondary) invasive breast tumours, which may become apparent long after exposure to RT. A meta-analysis by Akdeniz et al. did demonstrate a slightly increased risk of contralateral breast cancer after RT mainly in breast cancer patients treated <45 years of age [26].

Women <50 years diagnosed with primary DCIS between 1989 and 1998 had similar absolute late iIBC risk irrespective of treatment with BCS only or BCS + RT (Fig. 2). The SweDCIS trial neither showed a long-term beneficial effect of RT following BCS on iIBC risk in young women (<52 years) [16]. In our models, we split age at 50 years because the Dutch nationwide breast cancer screening starts at the age of 50 years and thus a diagnosis of primary DCIS in women <50 years is rarely based on breast screening. These women may present with a different type of DCIS, including more frequent symptomatic presentation (i.e. a lump), and/or may be diagnosed in the light of familial genetic susceptibility syndromes, which may be accompanied by an increased risk of iIBC. In addition, some studies [24, 27] showed that younger patients, in general, have a higher risk of invasive recurrences compared to older patients. However, Ryser et al. [3] did not find that iIBC risks were different between women aged <50 and ≥50 years, although this study was not powered to examine age differences. Therefore, we would be cautious against the interpretation that younger women benefit less from RT.

This large population-based DCIS cohort provides insight into the long-term risks of ipsilateral breast recurrences in women treated for DCIS. As DCIS is not a life-threatening disease, our ultimate goal should be to de-escalate treatment. There are ongoing efforts to determine whether molecular profiles of DCIS, such as Oncotype DX DCIS score [28] or DCISionRT signature [29], could support the selection of women in whom RT could be safely omitted. Furthermore, three ongoing clinical trials (LORIS [30], LORD [31] and COMET [32] trials) currently randomise between active surveillance and conventional treatment to omit therapy for women with low-risk DCIS. Understanding the dynamics of long-term residual breast cancer risk following treatment of DCIS contributes to the understanding of this disease and finally to reducing overtreatment.

## Supplementary information

Supplementary file

Reproducibility checklist

## Data Availability

The data generated and analysed during this study will be available from the corresponding author upon reasonable request.
