# Supplementary material for: Long-term risk of subsequent ipsilateral lesions after surgery with or without radiotherapy for ductal carcinoma in situ of the breast
Source: Br J Cancer. 2021 Aug 18;125(10):1443–9. doi: 10.1038/s41416-021-01496-6 (PMC8575990; doi:10.1038/s41416-021-01496-6)
Supplement: Supplementary file 1 — Supplementary file [file 41416_2021_1496_MOESM1_ESM.docx]

**Supplementary Material**

**Supplementary table 1** Absolute numbers of events and cumulative incidence, both split in periods of five years and split for initial DCIS treatment.

| **Type of Event** | **iDCIS** | | | | **iIBC** | | | | | |
| --- | --- | --- | --- | --- | --- | --- | --- | --- | --- | --- |
| **Treatment** | **BCS only** | | **BCS+RT** | | **BCS only** | | **BCS+RT** | | **Mastectomy** | |
|  | *Events (n)* | *Cum inc % (95%CI)* | *Events (n)* | *Cum inc % (95%CI)* | *Events (n)* | *Cum inc % (95%CI)* | *Events (n)* | *Cum inc % (95%CI)* | *Events (n)* | *Cum inc % (95%CI)* |
| 0-4 year | 258 | 10.0 (8.9-11.3) | 84 | 3.3 (2.6-4.0) | 173 | 6.9 (5.9-7.9) | 46 | 1.8 (1.4-2.4) | 33 | 0.7 (0.5-1.0) |
| 0-9 year | 333 | 13.0 (11.8-14.4) | 118 | 4.6 (3.9-5.5) | 315 | 13.9 (11.7-14.3) | 131 | 5.2 (4.4-6.2) | 55 | 1.1 (0.9-1.5) |
| 0-14 year | 349 | 13.7 (12.4-15.1) | 137 | 5.5 (4.7-6.5) | 390 | 16.3 (14.9-17.9) | 207 | 9.0 (7.9-10.3) | 79 | 1.6 (1.3-2.0) |
| 0-19 year | 352 | 13.9 (12.6-15.3) | 144 | 6.7 (5.5-8.1) | 432 | 19.1 (17.5-20.8) | 231 | 12.1 (10.5-14.0) | 87 | 1.9 ( 1.6-2.4) |
| 0-24 year | 352 | 13.9 (12.6-15.3) | 145 | 7.0 (5.7-8.6) | 444 | 21.6 (19.3-24.0) | 239 | 16.6 (13.4-20.6) | 89 | 2.2 (1.7-2.8) |
|  |  |  |  |  |  |  |  |  |  |  |
| 0 - 4 year | 258 | 10.0 (8.9-11.3) | 84 | 3.3 (2.6-4.0) | 173 | 6.9 (5.9-7.9) | 46 | 1.8 (1.4-2.4) | 33 | 0.7 (0.5-0.9) |
| 5 - 9 year | 75 | 3.6 (2.9-4.5) | 34 | 1.4 (1.0-2.0) | 142 | 7.0 (6.0-8.2) | 85 | 3.6 (2.9-4.4) | 22 | 0.5 (0.3-0.7) |
| 10 - 14 year | 16 | 0.9 (0.6-1.5) | 19 | 1.1 (0.7-1.7) | 75 | 4.7 (3.7-5.8) | 76 | 4.4 (3.5-5.5) | 24 | 0.6 (0.4-0.9) |
| 15 - 19 year | 3 | 0.3 (0.1-1.0) | 7 | 1.5 (0.7-3.2) | 42 | 4.4 (3.3-6.0) | 24 | 4.2 (2.8-6.4) | 8 | 0.4 (0.2-0.7) |
| 20 - 24 year | 0 | NA | 1 | NA | 12 | 5.1 (2.6-9.9) | 8 | 7.4 (3.6-15.0) | 2 | 0.4 (0.09-1.6) |

iIBC denotes ipsilateral invasive breast cancer; iDCIS: ipsilateral ductal carcinoma in situ; BCS: breast conserving surgery; RT: radiotherapy; n: number; Cum inc: cumulative incidence; 95%CI: 95% confidence interval.

**Supplementary table 2** Multivariable Cox analysis for iDCIS for patient diagnosed from 1999 – 2004.

|  |  | **iDCIS** |
| --- | --- | --- |
|  |  | **HR (95%CI)** |
| Treatment | BCS+RT | Ref |
|  | BCS only | 3.2 (2.3-4.4) |
| Age | <50 | 0.6 (0.3-1.0) |
|  | ≥50 | Ref |
| Grade* | Low (1) | 0.5 (0.3-0.8) |
|  | Intermediate (2) | Ref |
|  | High (3) | 1.2 (0.9-1.8) |

*Patients with unknown grade were excluded (n=525).

**Age as primary time-scale and adjusted for age at DCIS diagnosis (<50 vs ≥50).

HR denotes hazard ratio; iDCIS: ipsilateral ductal carcinoma in situ; 95%CI 95% confidence interval; Ref: reference; BCS: breast conserving surgery; RT: radiotherapy.

**Supplementary table 3** Multivariate Cox analysis to estimate the association of treatment with the risk of subsequent ipsilateral ductal carcinoma in situ (iDCIS) with follow-up from 3 months after DCIS diagnosis and from 6 months upwards.

|  |  |  | **iDCIS follow-up>3months** | | **iDCIS follow-up>6 months** | |
| --- | --- | --- | --- | --- | --- | --- |
| **Age at DCIS (years)** | **Time since DCIS (years)** | **Treatment** | **Events (N) / at risk (N)** | **HR** (**95%CI)** | **Events (N) / at risk (N)** | **HR** (**95%CI)** |
| <50  (N= 912) | 0-5 | BCS+RT | 15 / 457 | Ref | 12 / 454 | Ref |
|  |  | BCS only | 57 / 474 | 3.2 (1.6-6.6) | 45 / 462 | 4.9 (2.7-10.1) |
|  | 5-10 | BCS+RT | 8 / 419 | Ref | 8 / 419 | Ref |
|  |  | BCS only | 12 / 386 | 2.5 (1.1-5.3) | 12 / 386 | 3.6 (1.6-7.8) |
|  | ≥10 | BCS+RT | 17 / 383 | Ref | 17 / 283 | Ref |
|  |  | BCS only | 4 / 353 | 0.7 (0.3-1.5) | 4 / 353 | 1.0 (0.4-2.2) |
| ≥ 50  (N= 4239) | 0-5 | BCS+RT | 69 /2147 | Ref | 55 / 2133 | Ref |
|  |  | BCS only | 201 / 2173 | 3.6 (2.6-4.8) | 172 / 2144 | 3.9 (2.8-5.3) |
|  | 5-10 | BCS+RT | 26 / 1975 | Ref | 26 / 1975 | Ref |
|  |  | BCS only | 63 / 1701 | 2.7 (1.8-4.1) | 63 / 1701 | 2.8(1.8-4.3) |
|  | ≥10 | BCS+RT | 10 / 1769 | Ref | 10 / 1769 | Ref |
|  |  | BCS only | 15 / 1436 | 0.7 (0.4-1.3) | 15 / 1436 | 0.8 (0.4-1.4) |

Information regarding mastectomy treatment was not available for iDCIS.

(Attained) age as primary time-scale, adjusted for period of initial DCIS diagnosis (1989-1998 vs 1999-2004) and age at DCIS diagnosis (<50 vs ≥50) including an age-treatment interaction term.

HR denotes hazard ratio; 95%CI: 95% confidence interval; Ref: Reference category; BCS: Breast conserving surgery; RT: radiotherapy; iDCIS: ipsilateral ductal carcinoma in situ; iIBC ipsilateral invasive breast cancer; DCIS: ductal carcinoma in situ.

**Supplementary table 4** Multivariable Cox analysis for iIBC for patient diagnosed from 1999 – 2004 with and without including grade.

|  |  |  | **Model without grade** | **Model including grade** |
| --- | --- | --- | --- | --- |
| **Age at DCIS (years)** | **Time since DCIS (years)** | **Treatment** | **HR (95%CI)** | **HR (95%CI)** |
| <50 | 0-5 | BCS+RT | Ref | Ref |
|  |  | BCS only | 2.7 (1.2-6.1) | 2.8 (1.2-6.4) |
|  |  | Mastectomy | 0.5 (0.2-1.4) | 0.5 (0.2-1.4) |
|  | 5-10 | BCS+RT | Ref | Ref |
|  |  | BCS only | 1.8 (0.9-3.5) | 1.8 (0.9-3.7) |
|  |  | Mastectomy | 0.2 (0.1-0.6) | 0.2 (0.1-0.6) |
|  | ≥10 | BCS+RT | Ref | Ref |
|  |  | BCS only | 0.8 (0.4-1.7) | 0.8 (0.4-1.8) |
|  |  | Mastectomy | 0.2 (0.1-0.5) | 0.2 (0.1-0.6) |
| ≥50 | 0-5 | BCS+RT | Ref | Ref |
|  |  | BCS only | 4.2 (2.4-7.4) | 4.2 (2.4-7.6) |
|  |  | Mastectomy | 0.2 (0.1-0.4) | 0.2 (0.1-0.4) |
|  | 5-10 | BCS+RT | Ref | Ref |
|  |  | BCS only | 2.7 (1.8-4.2) | 2.7 (1.8-4.3) |
|  |  | Mastectomy | 0.1 (0.0-0.2) | 0.1 (0.0-0.2) |
|  | ≥10 | BCS+RT | Ref | Ref |
|  |  | BCS only | 1.3 (0.7-2.1) | 1.3 (0.8-2.2) |
|  |  | Mastectomy | 0.1 (0.0-0.2) | 0.1 (0.0-0.2) |
| Grade* |  | Low (1) | - | 0.9 (0.6-1.2) |
|  |  | Intermediate (2) | - | Ref |
|  |  | High (3) | - | 0.9 (0.7-1.2) |

*Patients with unknown grade were excluded (n=867)

** Age as primary time-scale, including a time-treatment interaction term and an age-treatment interaction term (p_interaction_=0.002), adjusted for age at DCIS diagnosis (<50 vs ≥50)

HR denotes hazard ratio; iDCIS: ipsilateral ductal carcinoma in situ; 95%CI: 95% confidence interval; Ref: reference; BCS: breast conserving surgery; RT: radiotherapy; DCIS ductal carcinoma in situ

**Supplementary table 5** Multivariate Cox analysis to estimate the association of treatment with the risk of subsequent ipsilateral ductal carcinoma in situ (iDCIS) and ipsilateral invasive breast cancer (iIBC) only including <70 years at DCIS diagnosis.

|  |  |  | **iDCIS** | | **iIBC** | |
| --- | --- | --- | --- | --- | --- | --- |
| **Age at DCIS (years)** | **Time since DCIS (years)** | **Treatment** | **Events (N) / at risk (N)** | **HR** (**95%CI)** | **Events (N) / at risk (N)** | **HR** (**95%CI)** |
| <50  (N=2143) | 0-5 | BCS+RT | 15 / 457 | Ref | 17 / 457 | Ref |
|  |  | BCS only | 57 / 474 | 3.5 (1.7-7.4) | 33 / 474 | 2.2 (1.4-3.4) |
|  |  | Mastectomy^a^ | - | - | 19 / 1212 | 0.4 (0.2-0.7) |
|  | 5-10 | BCS+RT | 8 / 419 | Ref | 22 / 412 | Ref |
|  |  | BCS only | 12 / 386 | 2.5 (1.1-5.5) | 23 / 379 | 1.0 (0.7-1.5) |
|  |  | Mastectomy^a^ | - | - | 13 / 1161 | 0.1 (0.1-0.2) |
|  | ≥10 | BCS+RT | 17 / 383 | Ref | 38 / 363 | Ref |
|  |  | BCS only | 4 / 353 | 0.6 (0.3-1.4) | 36 / 331 | 0.6 (0.4-0.9) |
|  |  | Mastectomy^a^ | - | - | 17 / 1108 | 0.1 (0.1-0.2) |
| 50-69  (N=6326) | 0-5 | BCS+RT | 58 / 1820 | Ref | 24 / 1820 | Ref |
|  |  | BCS only | 176 / 1721 | 3.8 (2.8-5.2) | 100 / 1721 | 3.6 (2.7-4.8) |
|  |  | Mastectomy^a^ | - | - | 11 / 2785 | 0.3 (0.2-0.5) |
|  | 5-10 | BCS+RT | 23 / 1699 | Ref | 60 / 1703 | Ref |
|  |  | BCS only | 56 / 1391 | 2.7 (1.7-4.2) | 99 / 1383 | 1.9 (1.4-2.6) |
|  |  | Mastectomy^a^ | - | - | 6 / 2671 | 0.1(0.1-0.2) |
|  | ≥10 | BCS+RT | 10 / 1546 | Ref | 64 / 1527 | Ref |
|  |  | BCS only | 13 / 1232 | 0.7 (0.4-1.2) | 80 / 1168 | 1.1 (0.9-1.5) |
|  |  | Mastectomy^a^ | - | - | 17 / 2501 | 0.1 (0.1-0.2) |

^a^Information regarding mastectomy treatment was not available for iDCIS.

(Attained) age as primary time-scale, adjusted for period of initial DCIS diagnosis (1989-1998 vs 1999-2004) and age at DCIS diagnosis (<50 vs ≥50) including an age-treatment interaction term.

HR denotes hazard ratio; 95%CI: 95% confidence interval; Ref: Reference category; BCS: Breast conserving surgery; RT: radiotherapy; iDCIS: ipsilateral ductal carcinoma in situ; iIBC ipsilateral invasive breast cancer; DCIS: ductal carcinoma in situ.

**Supplementary figure 1** Cumulative incidence of A) in situ and invasive recurrences, B) in situ recurrences only, C) invasive recurrences only including the competing risk (death) at 10 and 20 years following DCIS diagnosis.
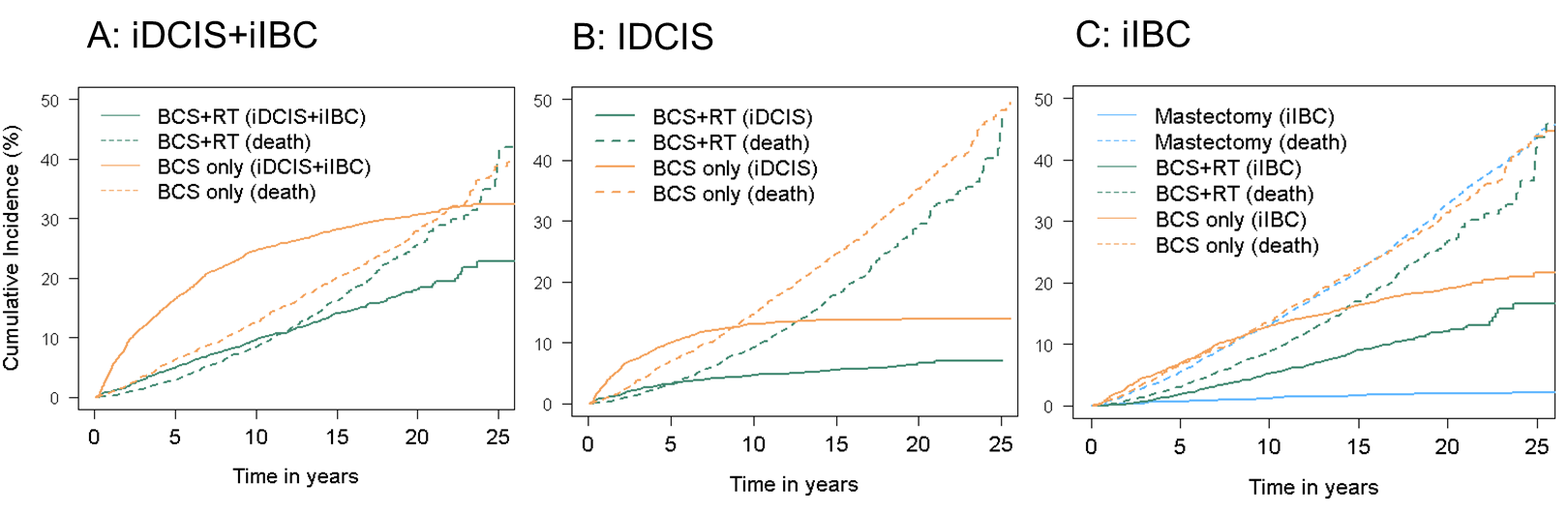


|  | **iDCIS+iIBC** | | **iDCIS** | | **iIBC** | | |
| --- | --- | --- | --- | --- | --- | --- | --- |
| **Treatment** | **BCS only**  *% (95%CI)* | **BCS+RT**  *% (95%CI)* | **BCS only**  *% (95%CI)* | **BCS+RT**  *% (95%CI)* | **BCS only**  *% (95%CI)* | **BCS+RT**  *% (95%CI)* | **Mastectomy**  *% (95%CI)* |
| *Cumulative incidence of event of interest* | | | | | | | |
| 0-9 year | 24.6 (23.0-26.3) | 9.6 (8.6-10.8) | 13.0 (11.8-14.4) | 4.6 (3.9-5.5) | 13.9 (11.7-14.3) | 5.2 (4.4-6.2) | 1.1 (0.9-1.5) |
| 0-19 year | 30.6 (28.9-32.6) | 18.2 (16.3-20.3) | 13.9 (12.6-15.3) | 6.7 (5.5-8.1) | 19.1 (17.5-20.8) | 12.1 (10.5-14.0) | 1.9 ( 1.6-2.4) |
| *Cumulative incidence of competing risk (death)** | | | | | | | |
| 0-9 year | 12.6 (11.4-13.9) | 8.4 (7.4-9.6) | 14.7 (13.4-16.1) | 9.2 (8.1-10.4) | 13.7 (13.4-16.1) | 8.7 (8.2-10.4) | 13.2 (12.3-14.2) |
| 0-19 year | 27.9 (26.0-29.9) | 25.7 (23.3-28.4) | 35.2 (33.2-37.4) | 29.2 (26.6-32.1) | 31.4 (29.3-33.6) | 26.8 (26.7-32.0) | 32.7 (31.3-34.3) |

*Death in absence of iDCIS and/or iIBC.

iIBC denotes ipsilateral invasive breast cancer; iDCIS: ipsilateral ductal carcinoma in situ; BCS: breast conserving surgery; RT: radiotherapy; 95%CI: 95% confidence interval.

**Grand Challenge PRECISION Consortium Steering Group:**

Jelle Wesseling (Netherlands Cancer Institute, Amsterdam, The Netherlands), Alastair Thompson (Baylor College of Medicine, Houston, Texas, USA), Serena Nik-Zainal (University of Cambridge, Cambridge, UK), Elinor J. Sawyer (King’s College London, London, UK), Helen Davies (University of Cambridge, Cambridge, UK), Andrew Futreal (MD Anderson Cancer Center, Houston, USA), Nicholas Navin (MD Anderson Cancer Center, Houston, USA), E. Shelley Hwang (Duke University School of Medicine, Durham, NC, USA), Jos Jonkers (Netherlands Cancer Institute, Amsterdam, The Netherlands), Jacco van Rheenen (Netherlands Cancer Institute, Amsterdam, The Netherlands), Fariba Behbod (Kansas University Medical Center, Kansas, USA), Esther H. Lips (Netherlands Cancer Institute, Amsterdam, The Netherlands), Marjanka Schmidt (Netherlands Cancer Institute, Amsterdam, The Netherlands), Lodewyk F.A. Wessels (Netherlands Cancer Institute, Amsterdam, The Netherlands), Daniel Rea (University of Birmingham, Birmingham, UK), Proteeti Bhattacharjee (Netherlands Cancer Institute, Amsterdam, The Netherlands), Hilary Stobart (Independent Cancer Patients' Voice, UK), Deborah Collyar (Patient Advocates in Research, USA), Donna Pinto (dcis411, USA), Ellen Verschuur (Borstkanker Vereniging Nederland, The Netherlands), Marja van Oirsouw (Borstkanker Vereniging Nederland, The Netherlands)
